# Supplementary material for: Identification of photoperiod-induced specific miRNAs in the adrenal glands of Sunite sheep (Ovis aries)
Source: Front Vet Sci. 2022 Jul 22;9:888207. doi: 10.3389/fvets.2022.888207 (PMC9354845; doi:10.3389/fvets.2022.888207)
Supplement: Supplementary file 1 [file Data_Sheet_1.ZIP › Supplementary file/miRDeep2 file.zip!/miRDeep2 file/Know_Structure/SP42A2/oar-mir-495.pdf]

miRBase precursor : oar-mir-495
Total read count : 18952
oar-miR-495-5p read count : 120
oar-miR-495-3p read count : 18810
remaining reads : 22

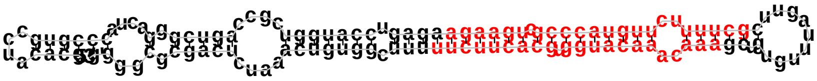

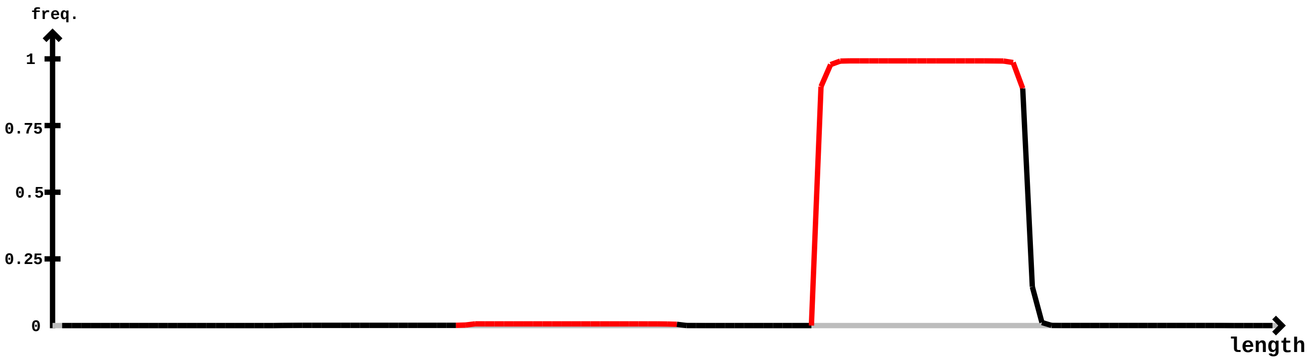


```
                                         oar-miR-495-3p
                                  oar-miR-495-5p
5'- gcacauccgugcccaucagggcugaccgcugguaccugagaagaagucgcccauguucuuuucgcuugauuugugucgaaacaaacauggugcacuucuuuuucggugucaaaucucagcgcgggg   -3'   exp
    ((((....))))((....(.(((((....(((((((.((((((((((.(((((((((..((((((.........).)))))..))))))).))))))))))))).)))))))....))))).)..))   reads   mm
    ......................ugaccgcugguaccugagaa....................................................................................   8       0
    ......................ugaccgcugguaccugagaag...................................................................................   1       0
    .......................gaccgcugguaccugagaa....................................................................................   5       0
    ........................accgcugguaccugagaa....................................................................................   3       0
    .........................................agaagucgcccauguucuu..................................................................   1       0
    .........................................agaagucgcccauguucuuu.................................................................   1       0
    .........................................agaagucgcccauguucuuuu................................................................   3       0
    .........................................agaagucgcccauguucuuuuc...............................................................   5       0
    .........................................agaagucgcccauguucuuuucg..............................................................   10      0
    ..........................................gaagucgcccauguucuu..................................................................   1       0
    ..........................................gaagucgcccauguucuuuu................................................................   4       0
    ..........................................gaagucgcccauguucuuuuc...............................................................   15      0
    ..........................................gaagucgcccauguucuuuucg..............................................................   77      0
    ..........................................gaagucgcccauguucuuuucgc.............................................................   1       0
    ..........................................gaagucgcccauguucuuuucgcu............................................................   2       0
    ..............................................................................aaacaaacauggugcacu..............................   2       0
    ..............................................................................aaacaaacauggugcacuA.............................   2       1
    ..............................................................................aaacaaacauggugcacuu.............................   6       0
    ..............................................................................aaacaaacauggugcacuuc............................   103     0
    ..............................................................................aaacaaacauggugcacuucu...........................   1696    0
    ..............................................................................aaacaaacauggugcacuucuu..........................   13401   0
    ..............................................................................aaacaaacauggugcacuucuuu.........................   1725    0
    ..............................................................................aaacaaacauggugcacuucuuuu........................   46      0
    ...............................................................................aacaaacauggugcacu..............................   1       0
    ...............................................................................aacaaacauggugcacuu.............................   1       0
    ...............................................................................aacaaacauggugcacuuc............................   1       0
    ...............................................................................aacaaacauggugcacuucu...........................   133     0
    ...............................................................................aacaaacauggugcacuucuu..........................   670     0
    ...............................................................................aacaaacauggugcacuucuuu.........................   726     0
    ...............................................................................aacaaacauggugcacuucuuuu........................   34      0
    ................................................................................acaaacauggugcacuuc............................   1       0
    ................................................................................acaaacauggugcacuucu...........................   13      0
    ................................................................................acaaacauggugcacuucuu..........................   26      0
    ................................................................................acaaacauggugcacuucuuu.........................   88      0
```

```
                                                                                    oar-miR-495-3p
                                         oar-miR-495-5p
gcacauccgugcccaucagggcugaccgcugguaccugagaagaagucgcccauguucuuucgcuugauuugugucgaaacaaacauggugcacuucuuuuucggugucaaaucucagcgcgggg

...............................................................................acaaacauggugcacuucuuuu........................    118    0
...............................................................................acaaacauggugcacuucuuuuu.......................    1      0
................................................................................caaacauggugcacuucu...........................    1      0
................................................................................caaacauggugcacuucuu..........................    1      0
................................................................................caaacauggugcacuucuuu.........................    1      0
................................................................................caaacauggugcacuucuuuu........................    10     0
................................................................................caaacauggugcacuucuuuuu.......................    3      0
...................................................................................................uuucggugucaaaucucagc......    1      0
...................................................................................................uuucggugucaaaucucagcg.....    4      0
```
